# Cell salvage using the continuous autotransfusion device CATSmart – an observational bicenter technical evaluation

**DOI:** 10.1186/s12871-018-0651-0

**Published:** 2018-12-12

**Authors:** Simone Lindau, Madeline Kohlhaas, Michael Nosch, Suma Choorapoikayil, Kai Zacharowski, Patrick Meybohm

**Affiliations:** 1 Department of Anaesthesiology, Intensive Care Medicine and Pain Therapy, University Hospital Frankfurt, Theodor-Stern-Kai 7, 60590 Frankfurt am Main, Germany; 2 Department of Anaesthesiology, Intensive Care Medicine and Pain Therapy, Marienhospital Bottrop gGmbH, Bottrop, Germany

**Keywords:** Cell salvage, Auto transfusion, Hematocrit value

## Abstract

**Background:**

The use of cell salvage and autologous blood transfusion has become an important method of blood conservation. So far, there are no clinical data about the performance of the continuous autotransfusion device CATSmart.

**Methods:**

In total, 74 patients undergoing either cardiac or orthopedic surgery were included in this prospective, bicenter and observational technical evaluation to validate red cell separation process and washout quality of CATSmart. The target of red cell separation process was defined as a hematocrit value in the packed red cell unit of 55–75% and of washout quality of 80–100% removal ratio.

**Results:**

Hematocrit values measured by CATSmart and laboratory analysis were 78.5% [71.3%; 84.0%] and 73.7% [67.5%; 75.5%], respectively. Removal ratios for platelets 94.7% [88.2%; 96.7%], free hemoglobin 89.3% [85.2%; 94.9%], albumin 97.9% [96.6%; 98.5%], heparin 99.9% [99.9%; 100.0%], and potassium 92.5% [90.8%; 95.0%] were within the target range while removal of white blood cells was slightly worse 72.4% [57.9%; 87.3%].

**Conclusion:**

The new autotransfusion device enables sufficient red cell separation and washout quality.

## Background

Cell salvage is the process by which blood from the surgical field or wound drainages is collected, filtered, and washed to produce autologous blood for re-transfusion to the patient. With advances in washing and filtration technology, new cell salvage devices now provide a high quality blood product for re-infusion. Furthermore, the use of autologous blood is theoretically attractive as being less harmful than allogeneic red blood cells (RBC) [1]. A meta-analysis conducted by our group was published in 2016 and included 47 trials suggesting that cell salvage is efficacious in reducing the need for allogeneic RBC transfusion during surgery by 39% [1]. CATSmart is a continuous autotransfusion system equipped amongst others with an integrated hematocrit (Hct) sensor [2]. In addition, CATSmart uses an unique and fastest continuous red cell separation process based on Continuous Flow technology which guarantees an early access to RBC at any time during surgery with a reliable quality. CATSmart also combines clinical performance and ergonomic benefits [3]. The comparison of CATSmart with the predecessor using banked blood showed similar efficiency with regards to RBC recovery, plasma and fat elimination [4]. Until now, CATSmart has not yet been tested in clinical settings. Thus, we validated the red cell separation process and the washout quality of CATSmart in two independent cohorts of cardiac and orthopedic surgery.

## Methods

### Design

Two prospective and observational cohorts were assessed in the Marienhospital Bottrop and the University Hospital Frankfurt between January 2015 and September 2017. Data were merged for publication.

Patients (>= 18 years) undergoing elective orthopedic or cardiac surgery with expected blood loss > 10% of body blood volume were included. Exclusion criterion was pregnancy.

### Outcome

The primary outcomes were red cell separation performance and washout quality. Red cell separation performance was defined as sufficient if haematocrit (Hct) values of the autologous packed red cell (PRC) concentrate reached a target range between 55 and 75% according to the current German Guidelines [5]. Washout quality was defined as sufficient if the removal ratio (RR) reached target range between 80 and 100%. Washout quality was evaluated for potassium (K^+^), albumin (Alb), heparin (aXa), free hemoglobin (fHb), white blood cells (WBC), and platelets (Plt). Calculation of RRs was based on the following formula: RR [%] = [1-(V_PRC_ x {Sub}_PRC_)/(V_RES_ x {Sub}_RES_)] × 100 [%] (V_PRC_=PRC volume; V_RES_=Shed blood volume; {Sub}_PRC_ = concentration of a substance in PRC volume; {Sub}_RES_ = concentration of a substance in shed blood in reservoir).

### Procedures

After collection of a minimum of 400 ml shed blood during surgery, shed blood was processed by a CE-certified autotransfusion device (CATSmart®, Fresenius Medical, Germany). The shed blood was collected in a sterile reservoir, was processed in a continuous running centrifuge for red cell separation and washed using the smart wash mode (standard wash program, packed red cells output rate 20-40 ml/min). The product was a sterile bag filled with washed packed red cells for reinfusion into the patient. During this process all plasmatic and non-erythrocyte cellular components of the collected blood, and thus activated coagulation factors, products of fibrinolysis and cell trauma as well as the anticoagulant were removed. The Hct of the incoming shed blood and outgoing washed PRC is measured and visualized on the device screen, for information. To characterize Hct values, blood samples were taken from the CATSmart reservoir and the PRC concentrate, according established procedures before and after the washing step and analyzed in the central laboratory (LAB). Blood reservoir was manually homogenized before blood sampling.

### Statistical analysis

Data are provided as median, 25% quartile and 75% quartile when indicated, and a *p*-value of ≤0.05 was considered as statistically significant. RR of K^+^, Alb, aXa, fHb, WBC, and Plt were calculated for each patient. Correlation coefficient was determined by Spearman. Microsoft Excel 2010 was used for all statistical calculations. A minimum of 20 patients was defined as sufficient for this descriptive analysis.

## Results

**Fig. 1:**
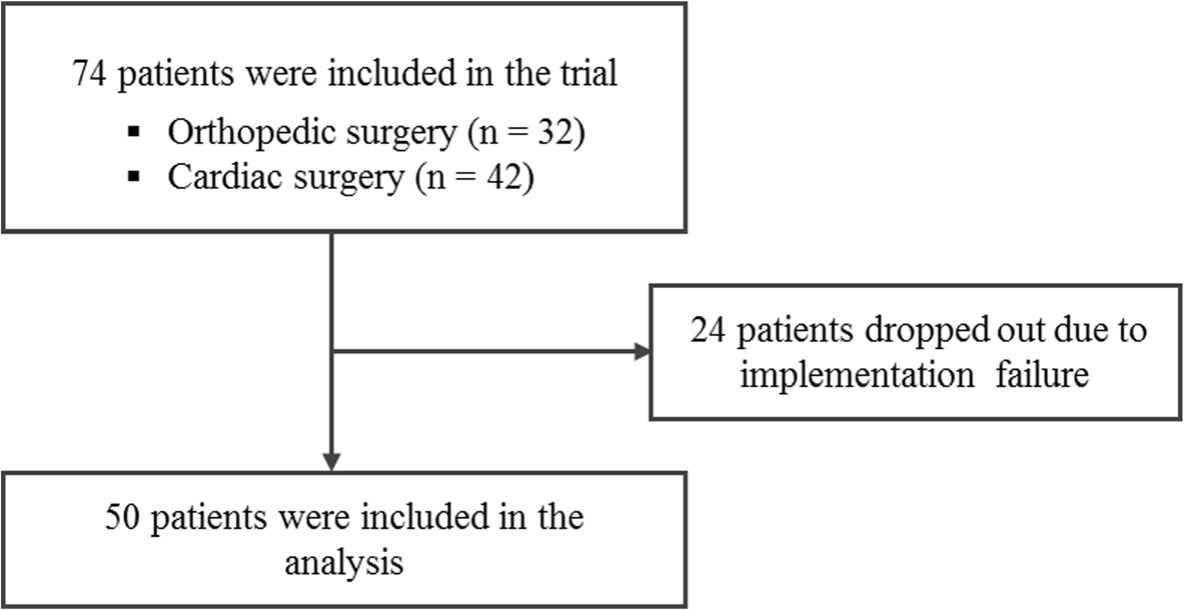
Flowchart of participants

A total of 74 patients were recruited of which 24 were excluded due to implementation failure of the new device. In total, 50 patients undergoing orthopedic (*n* = 32) and cardiac surgery (*n* = 18) were included in the final analysis (Fig. 1).

### Red cell separation performance

**Fig. 2:**
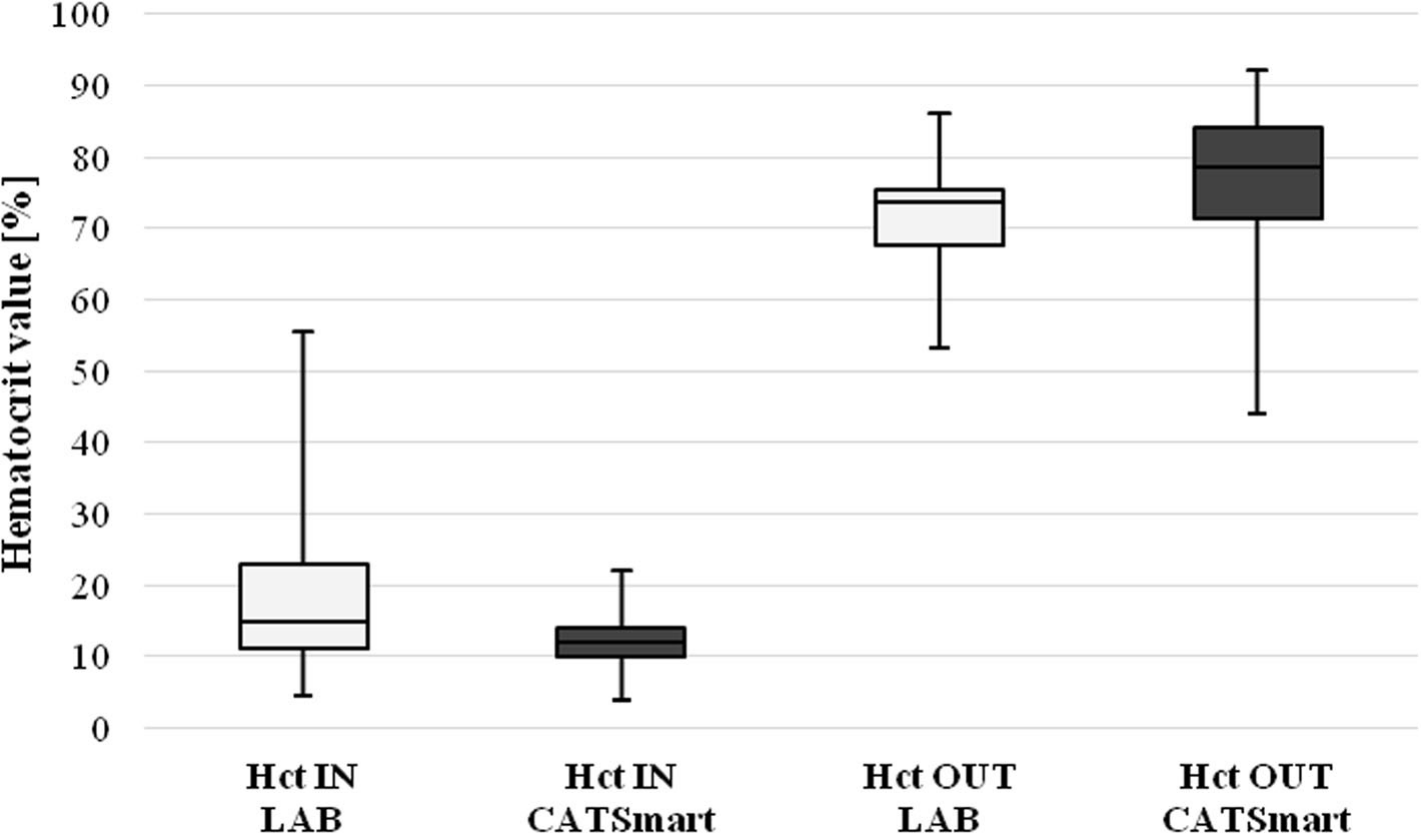
Red cell separation performance defined by hematocrit values. Data are presented as median [25%;75%]. Hematocrit (Hct) values are shown before (IN, shed blood) and after blood processing (OUT, packed red cells) by central laboratory (LAB) and CATSmart.

**Fig. 3:**
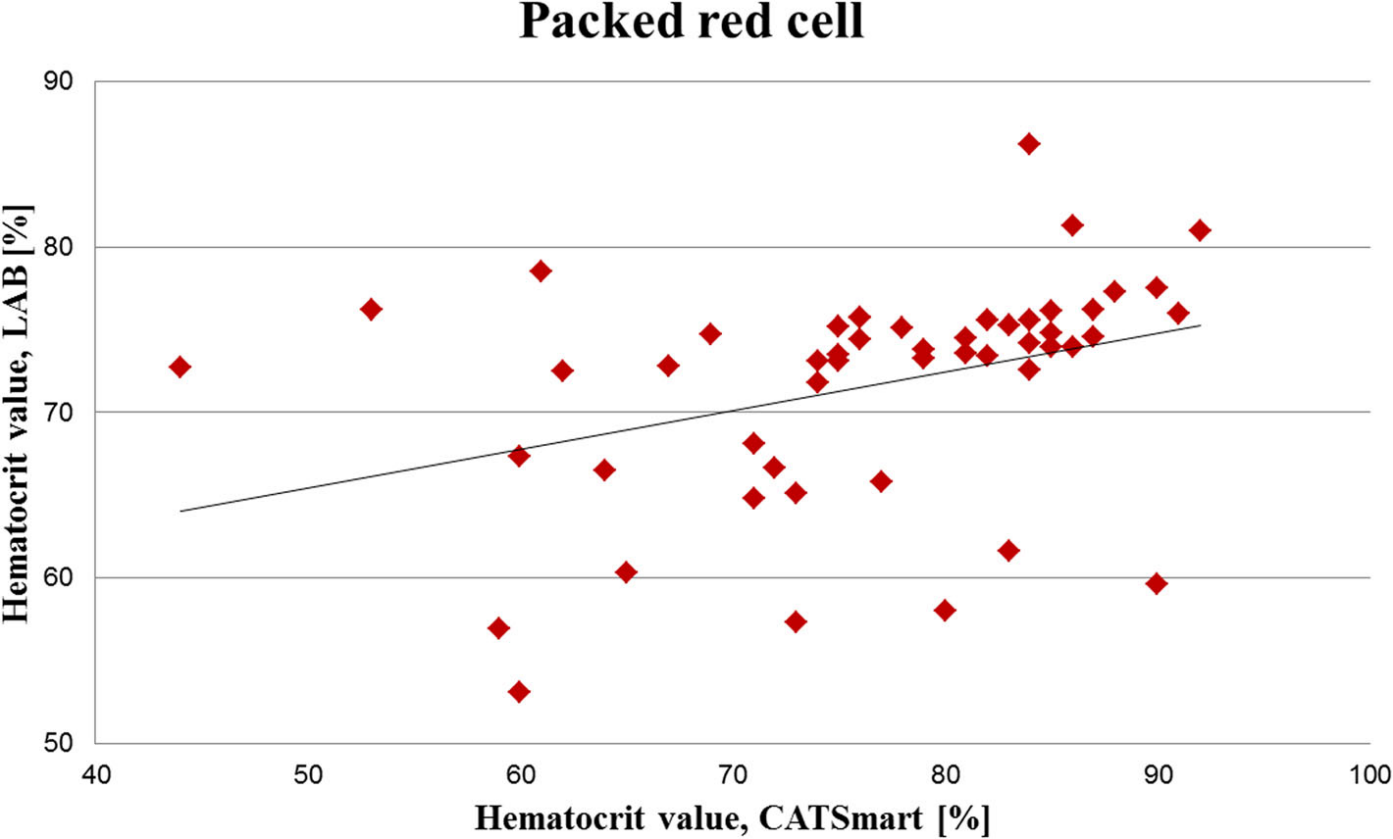
Hematocrit values in RBC concentrates. Hct values of the packed red cell concentrate measured by CATSmart and the laboratory (LAB) significantly correlated (*r* = 0.51; *p* ≤ 0.05).

Median ([25%;75%] Hct values in shed blood were 12.0% [10.0%; 14.0%], CATSmart) and 14.9% ([11.0%; 22.9%], LAB) (*p* ≤ 0.05) and in the PRC concentrate 78.5% ([71.3%; 84.0%], CATSmart) and 73.7% ([67.8%; 75.6%], LAB) (*p* ≤ 0.05) (Fig. 2). Hct values of the PRC concentrates measured by CATSmart and LAB significantly correlated (*r* = 0.51; *p* ≤ 0.05) (Fig. 3).

### Washout quality

**Fig. 4:**
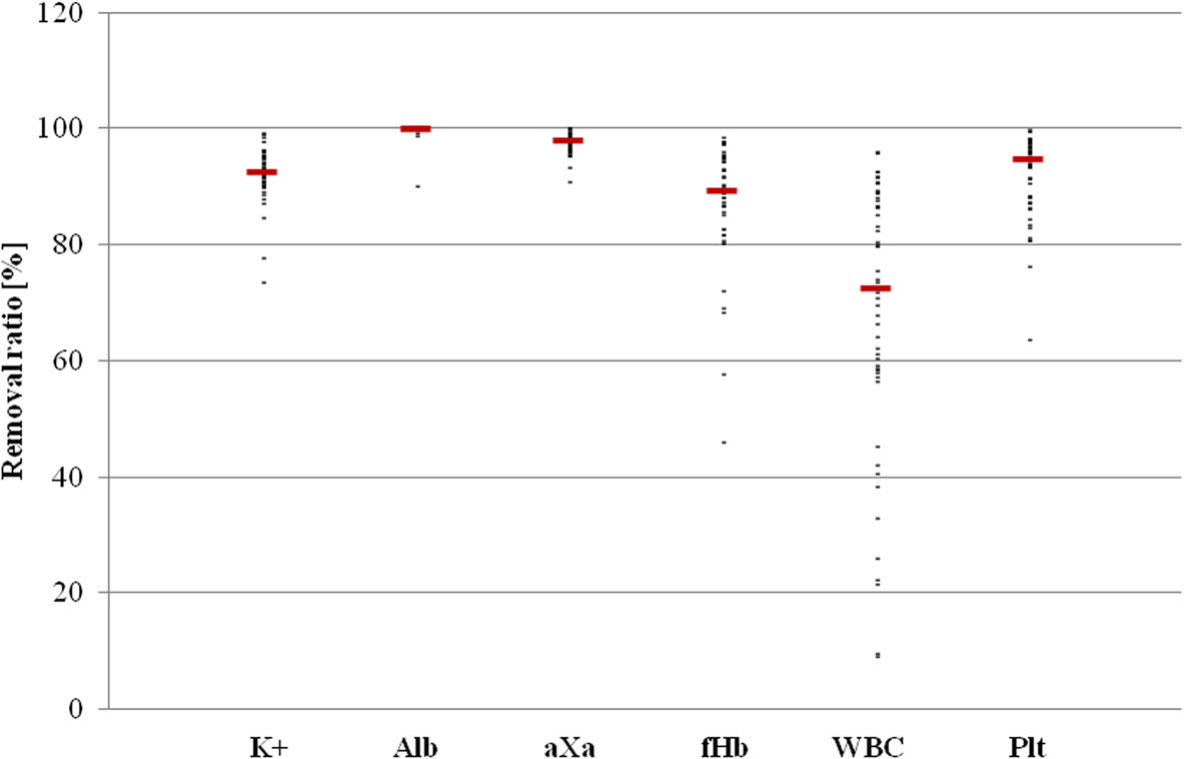
Washout quality defined by removal ratio (%) of multiple blood parameters for each of the 50 analyzed patients. WBC = white blood cells; Plt = platelets; Alb = albumin; fHb = free hemoglobin; aXa = heparin; K^+^ = potassium.

Individual values varied between 73.3–99.1% (median [25%;75%]; 92.5% [90.8%; 95.0%]) for K^+^, 90.7–100.0% (97.9% [96.6%; 98.5%]) for Alb, 90.0–100.0% (99.9% [99.9%; 100.0%]) for aXa, 45.8–98.5% (89.3% [85.2%; 94.9%]) for fHb, 9.0 and 95.9% (72.4% [57.9%; 87.3%]) for WBC, and 63.4–99.6% (94.7% [88.2%; 96.7%]) for Plt (Table 1, Fig. 4).

**Table 1:** Washout quality defined by removal ratio of blood parameters

Parameters	RR_median [25%;75%]_ (%)	RR_min_ (%)	RR_max_ (%)
K^+^ (mmol/l)	92.5 [90.8;95.0]	73.3	99.1
Alb (g/dl)	97.9 [96.6;98.5]	90.7	100.0
aXa (U/ml)	99.9 [99.9;100.0]	90.0	100.0
fHb (g/dl)	89.3 [85.2;94.9]	45.8	98.5
WBC (/nl)	72.4 [57.9;87.3]	9.0	95.9
Plt (/nl)	94.7 [88.2;96.7]	63.4	99.6

Data are presented as median [25%;75%], minimum (min) and maximum (max). *RR* Removal ratio, *K*^*+*^ potassium, *Alb* albumin, *aXa* heparin, *fHb* free hemoglobin, *WBC* white blood cells, *Plt* platelets

## Discussion

The importance of cell salvage and autologous red cell retransfusion has increased dramatically in recent years [6, 7]. This may be attributed to patient blood management (PBM) programs [8] which emphasize the benefits of cell salvage as an important tool to reduce surgery-related blood loss and allogeneic RBC transfusion rate [9]. A recent meta-analyses revealed that the use of cell recovery and autologous retransfusion is associated with a reduction in allogeneic RBC transfusion by up to 39%, decreased the risk of infection by 28% and shortened length of stay in hospital by 2.31 days [1]. Similar results were found in a meta-analysis conducted by Wang and colleagues [10]. The intraoperative use of cell salvage reduced the exposure of any allogeneic blood products by 37% and transfusion of RBCs by 40% in cardiac surgical patients.

Recently, CATSmart has been introduced into the market, as the next generation model of the continuous autotransfusion device C.A.T.S ^*plus*^. In an ex-vivo model, both devices demonstrated sufficient performance in terms of Hct, RBC recovery, elimination rates of protein, heparin and fat, and hemolysis rates [2, 4].

While some previous studies focused on in vitro validation, this report evaluates the efficacy of CATSmart in clinical settings and human blood. To assess red cell separation performance, we compared Hct values before and after blood processing using the CATSmart device and by laboratory analysis of the RBC concentrate. Hct values and RR were within the target ranges of 55–75% and 80–100% respectively. Interestingly, median Hct values measured by the device itself were even slightly above the target range 78.5% [71.3%; 84.0%] due to differences in measurements. While CATSmart assess Hct value during the washing process, laboratory analysis calculates Hct values by a single-point measurement from a blood sample taken directly before and after the washing process and insufficient homogenization of the blood reservoir might have influenced Hct levels. Overall elimination of quality parameters was within the target ranges. However, RR of WBC (72.4% [57.9%; 87.3%]) was slightly below the target range. Alberts and colleagues also found inadequate washout results with a WBC elimination rate of 34.7% [2]. Finally, our results are based on the standard wash program “smart wash mode”, therefore future studies should compare quality parameters using different program modes.

## Conclusion

The importance of cell salvage and autologous red cell re-transfusion increased dramatically during the last years. However, technical devices and systems are subject to constant change. Our results demonstrate that the CATSmart device shows sufficient red cell separation performance and washout quality.

## Abbreviations

{Sub}_PRC_: Concentration of a substance in PRC volume
{Sub}_RES_: Concentration of a substance in shed blood in reservoir
Alb: Albumin
aXa: Heparin
fHb: free hemoglobin
Hct: Hematocrit
K^+^: Potassium
LAB: Analyzed in the laboratory
Max: Maximum
Min: Minimum
PBM: Patient Blood Management
Plt: Platelets
PRC: Packed red cell
RBC: Red blood cells
RR: Removal ratio
V_PRC_: PRC volume
V_RES_: Shed blood volume
WBC: White blood cells
